# Supplementary material for: Low Bacterial Biomass in Human Pancreatic Cancer and Adjacent Normal Tissue
Source: Int J Mol Sci. 2024 Dec 27;26(1):140. doi: 10.3390/ijms26010140 (PMC11720454; doi:10.3390/ijms26010140)
Supplement: Supplementary file 1 [file ijms-26-00140-s001.zip › ijms-3366202-supplementary.pdf]

International Journal of
Molecular Sciences

MDPI

Supplementary Materials:

Low Bacterial Biomass in Human Pancreatic Cancer and Adjacent Normal Tissue

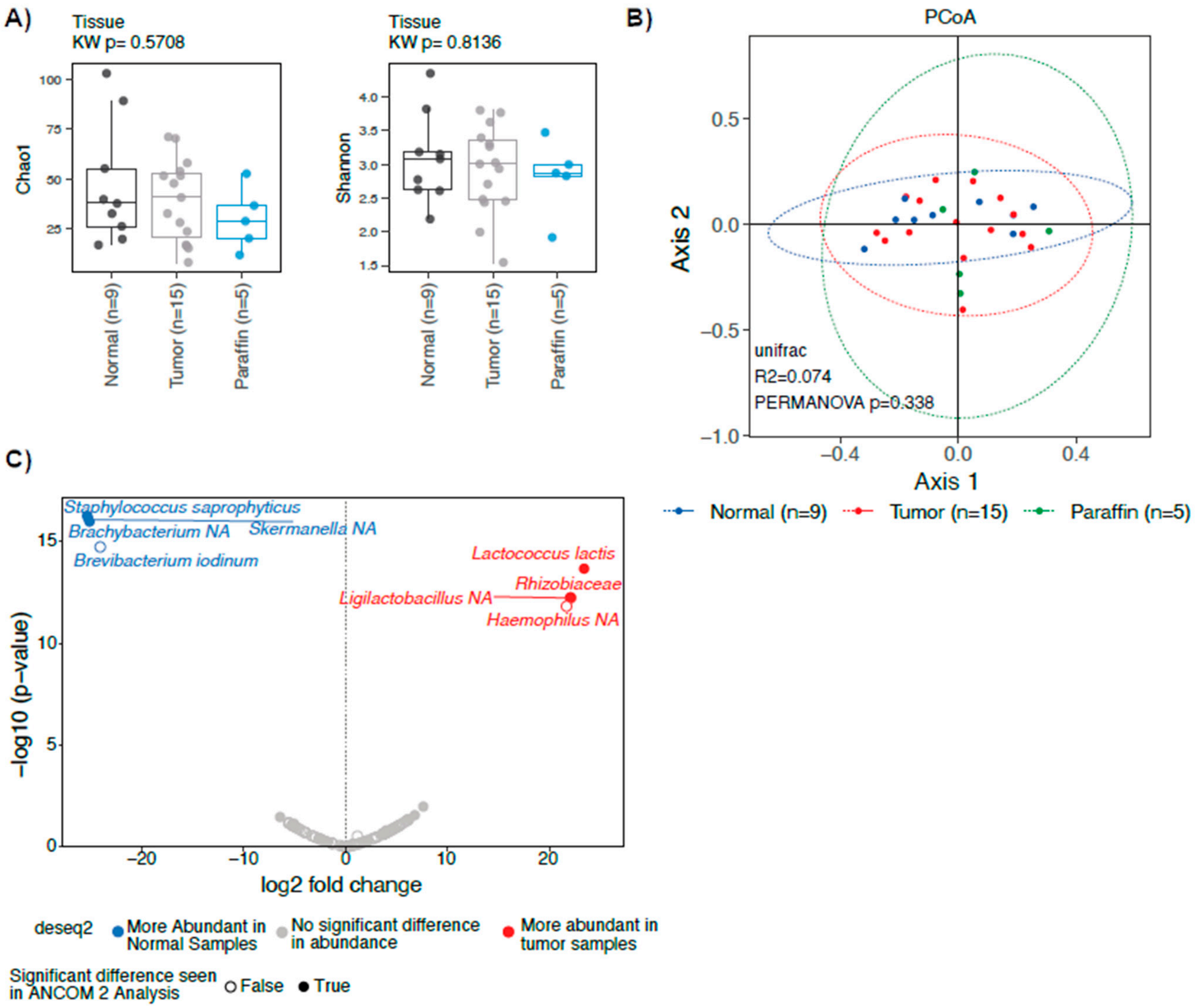

Figure S1. Microbial diversity in FFPE samples. **A**) Alpha-diversity indices: Chao1 (left) and Shannon (right) with Kruskal–Willis test results. **B**) PCoA plot showcasing microbial community structures based on Unifrac dissimilarity distances. The p-value and R^2 values were derived from a permutational multivariate analysis of variance (PERMANOVA) test. **C**) Differential abundance test results combining DESeq2 and ANCOM2 methodologies. Colored symbols represent taxa identified by DESeq2, while closed circles indicate taxa identified by ANCOM2.

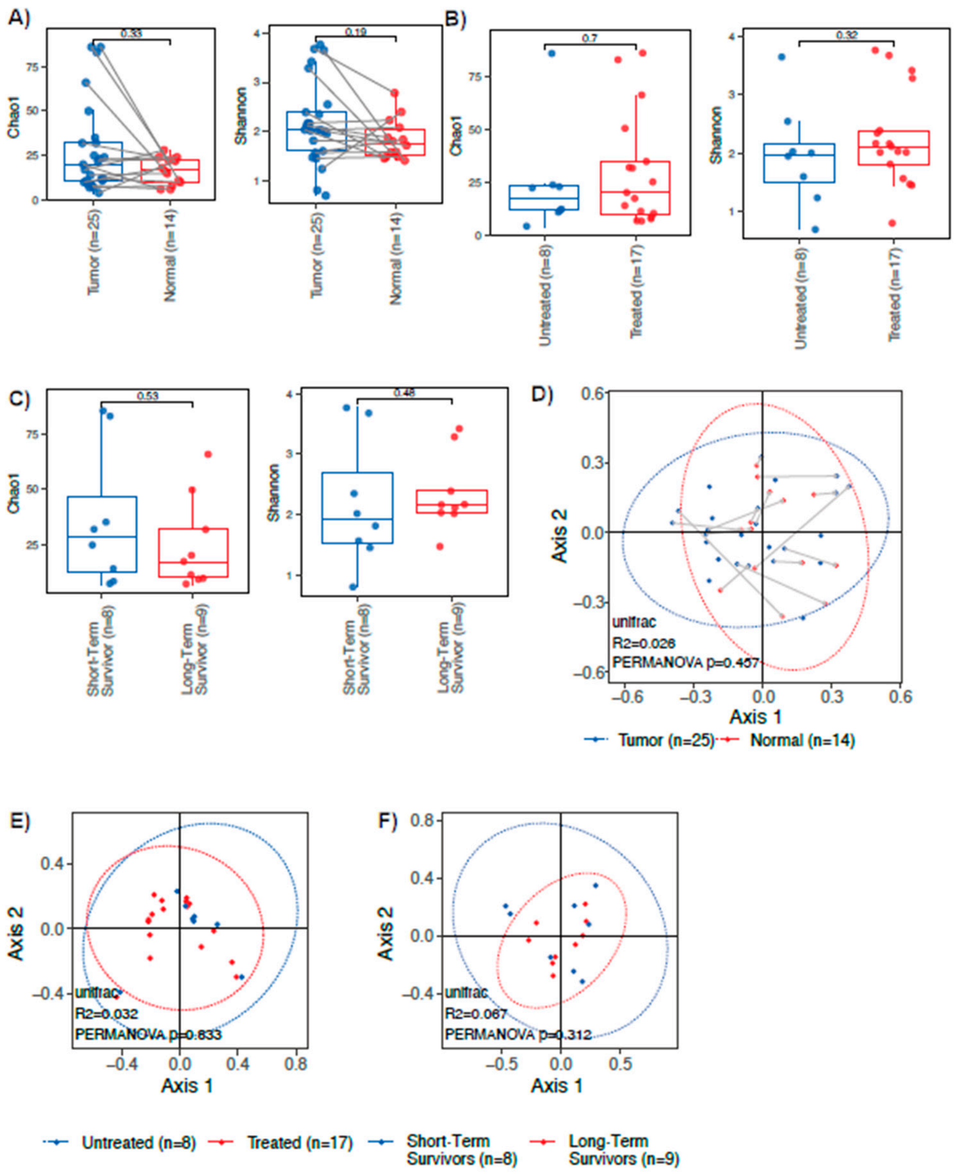

Figure S2. Microbial diversity in fresh frozen samples. **A–C**) Alpha-diversity indices: Chao1 (left) and Shannon (right) with Kruskal–Willis test results for: **A**) tissue type, **B**) neoadjuvant treatment with gemcitabine-based chemotherapy, and **C**) survival duration. **D-F**) PCoA plots based on Unifrac dissimilarity distances. The p-value and R^2 values were calculated using the PERMANOVA test for: **D**) tissue type, E) neoadjuvant treatment with gemcitabine-based chemotherapy, and F) survival duration. For tissue type, both Chao1 and Shannon indices displayed a decreasing trend from tumor to normal tissues, but without statistical significance (Chao1: p=0.33, Shannon: p=0.19). Regarding neoadjuvant gemcitabine treatment, an increasing trend was observed from untreated to treated groups, yet the differences were not statistically significant (Chao1: p=0.7, Shannon: p=0.32). For survival duration, Chao1 decreased (p=0.53) while Shannon increased (p=0.48) between short and long survival cohorts. Beta-diversity, visualized using PCoA plots with Unifrac dissimilarity distances, did not show clear clustering for any of the three groups, and PERMANOVA analysis confirmed the lack of significant differences (p-values: 0.457, 0.833, 0.312).

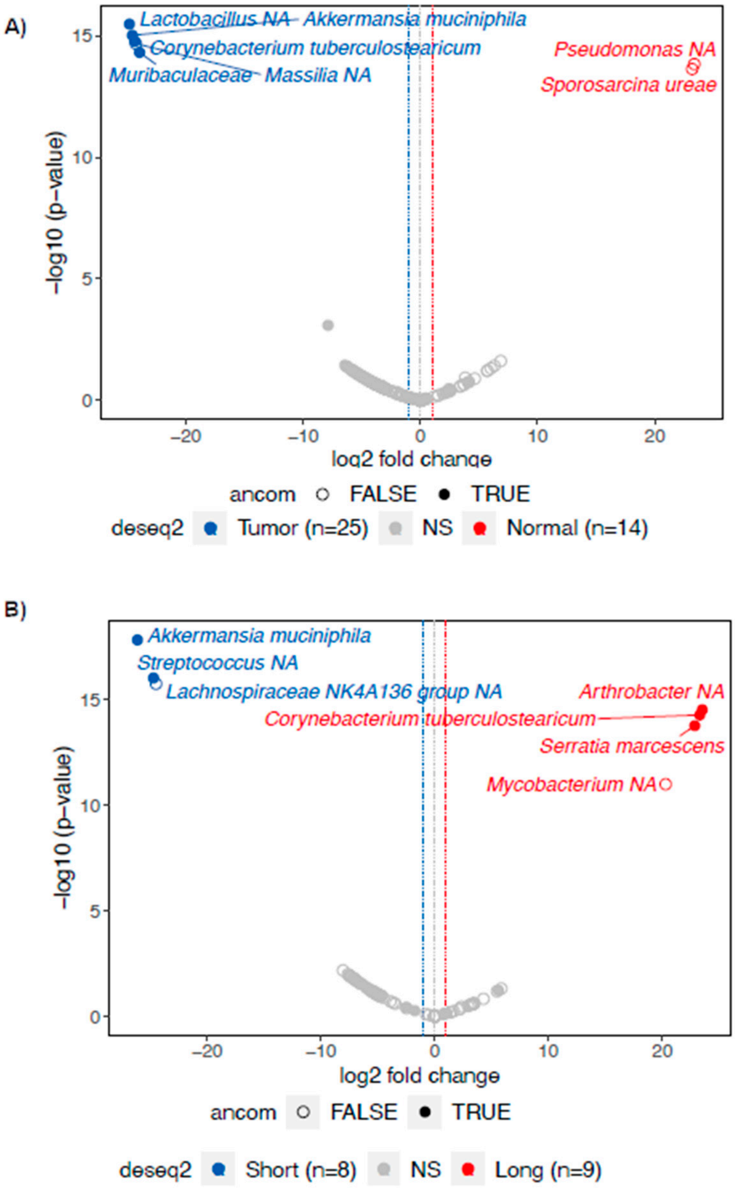

Figure S3. Differential abundance test results combining DESeq2 and ANCOM2 methodologies. Colored symbols represent taxa identified by DESeq2, while closed circles indicate taxa identified by ANCOM2 for: **A**) tissue type and **B**) survival duration.
